# Cardiac involvement in heavy and light chain amyloidosis: A case report and literature review

**DOI:** 10.1097/MD.0000000000017999

**Published:** 2019-11-15

**Authors:** Yukihiro Otaka, Yoichi Nakazato, Takaaki Tsutsui, Jun’ichi Tamura

**Affiliations:** a Department of General Medicine, Gunma University Graduate School of Medicine, Maebashi; b Kidney Disease and Dialysis Center; c Department of Pathology, Hidaka-kai Hidaka Hospital, Takasaki, Gunma, Japan.

**Keywords:** cardiac amyloidosis, conventional laboratory test, heavy and light chain amyloidosis, sex difference

## Abstract

**Introduction:**

Heavy and light chain amyloidosis is an extremely rare condition. There are few reports referring to the clinical impact of cardiac involvement in heavy and light chain amyloidosis, and the significance of myocardial impairment has not yet been completely explained.

**Patient concerns:**

A 66-year-old Japanese man was admitted to our hospital presenting with nephrotic syndrome and congestive heart failure.

**Diagnosis:**

Kidney and endoscopic gastric mucosal biopsy demonstrated congophilic hyalinization in most of the glomeruli and surrounding vessel walls, which were highly positive for immunoglobulin A and lambda. Finally, the patient was diagnosed as an atypical multiple myeloma with systemic heavy and light chain amyloidosis.

**Interventions:**

The patient was referred to hematology for further treatment and was moved to another hospital for the administration of chemotherapy using melphalan and dexamethasone.

**Outcomes:**

The patient was still alive after 15-month follow-up from the initial diagnosis.

**Conclusion:**

Initial screening and follow-up for cardiac involvement are important for heavy and light chain amyloidosis. Further investigation for the prognosis of heavy and light chain amyloidosis is required to improve the strategies of diagnosis and treatment options for patients with this disease.

## 1 Introduction

Amyloidosis is an uncommon condition characterized by the extracellular aggregation of insoluble fibrils resulting from sequential changes in the misfolding of certain proteins. To date, 36 precursor proteins and their amyloid variants have been identified.^[1]^ The most common form of systemic amyloidosis is immunoglobulin (Ig)-related amyloidosis, which mainly comprises monoclonal light chain amyloidosis (AL amyloidosis).^[2,3]^ AL amyloidosis accounts for approximately two-thirds of all systemic amyloidosis cases in developed countries.^[4]^ Among Ig-related amyloidosis, heavy and light chain amyloidosis (AHL amyloidosis) is exceptionally rare and is characterized by amyloid deposition derived from both heavy and light chains. AHL amyloidosis accounts for only 4.2% to 7.5% of Ig-related amyloidosis,^[2,3]^ and has never been included in the official International Society of Amyloidosis Fibril Protein Nomenclature List as a distinct precursor protein.^[1]^ Due to limited access to clinical data, little is known about the entities of AHL amyloidosis.

The most commonly involved organ in AL amyloidosis is the kidney, followed by the heart.^[4,5]^ Cardiac amyloidosis is associated with sudden cardiac death due to pulseless electrical activity following ventricular arrhythmias and is a poor prognostic factor in patients with AL amyloidosis.^[6,7]^ Additionally, it is thought that sudden death may be caused by following thromboembolic complications, bradyarrhythmias, and conduction system disorders.^[6]^ In contrast, there is little information about the risk of myocardial impairment in AHL amyloidosis. There is only one case series study of renal AHL with heavy chain amyloidosis (AH amyloidosis), the authors concluded that renal AHL/AH amyloidosis has less cardiac involvement than that in AL amyloidosis.^[8]^ However, there are few reports referring to the clinical impact of cardiac involvement in AHL amyloidosis, and the significance of myocardial impairment has not yet been completely explained.

Here, we add to the literature on AHL amyloidosis by describing a Japanese male case of AHL amyloidosis presenting with nephrotic syndrome and congestive heart failure. Written consent from the patient was obtained before submission. All presented materials have been fully anonymized to protect the individual's identity. In addition, we reveal new points of view on AHL amyloidosis *via* an analysis of the current literatures.

## 2 Case presentation

A 66-year-old Japanese man was consulted to our hospital due to the progression of dyspnea and edema in both legs, which had occurred during the previous week. He presented with a low-grade fever (37.4°C by armpit), productive cough and hypoxemia with 94% of percutaneous oxygen saturation in ambient air. He was an ex-smoker (10 cigarettes per day, for 6 years from 20 to 25 years of age), but had no particular past medical history or medications.

**Figure 1:**
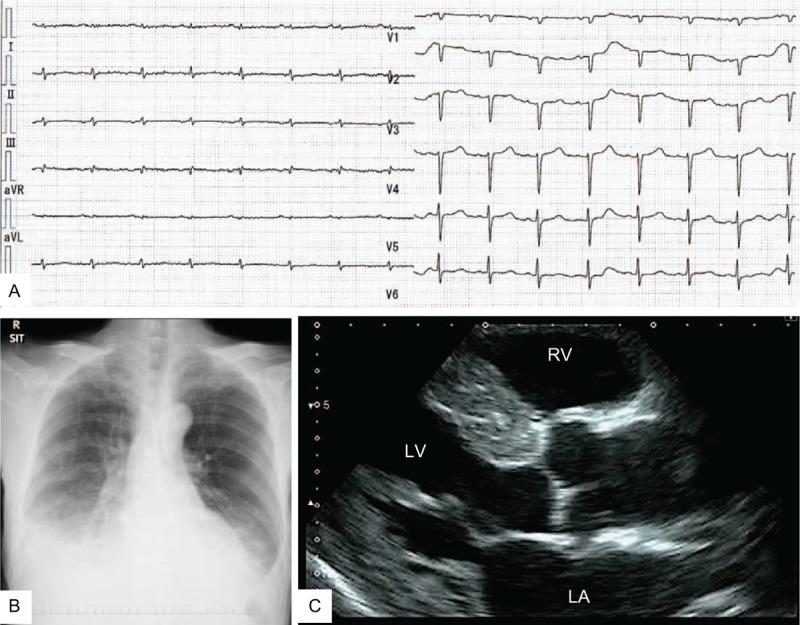
(A) Electrocardiography showing low voltage and a long corrected-QT (cQT) interval in the limb leads. The cQT was 489 ms. (B) Chest X-ray showing dilation of a cardiac shadow and bilateral pleural effusion. (C) Transthoracic echocardiography revealing left ventricular hypertrophy and high echoic granular spots at the septum. LA = left atrium; LV = left ventricle; RV = right ventricle.

The results of the initial laboratory tests showed hypoproteinemia, proteinuria, and moderate renal insufficiency (Table 1). On electrocardiography, low voltage and a long QT interval were detected (Fig. 1A). A chest x-ray showed dilation of a cardiac shadow and bilateral pleural effusion (Fig. 1B). Echocardiography revealed left ventricular hypertrophy (mean wall thickness, 15 mm) and granular high echoic spots at the septum (Fig. 1C). His plasma brain natriuretic peptide level was increased (448.7 pg/mL; normal range, < 18.4 pg/mL). These typical observations are compatible with amyloid cardiomyopathy.

**Table 1:**
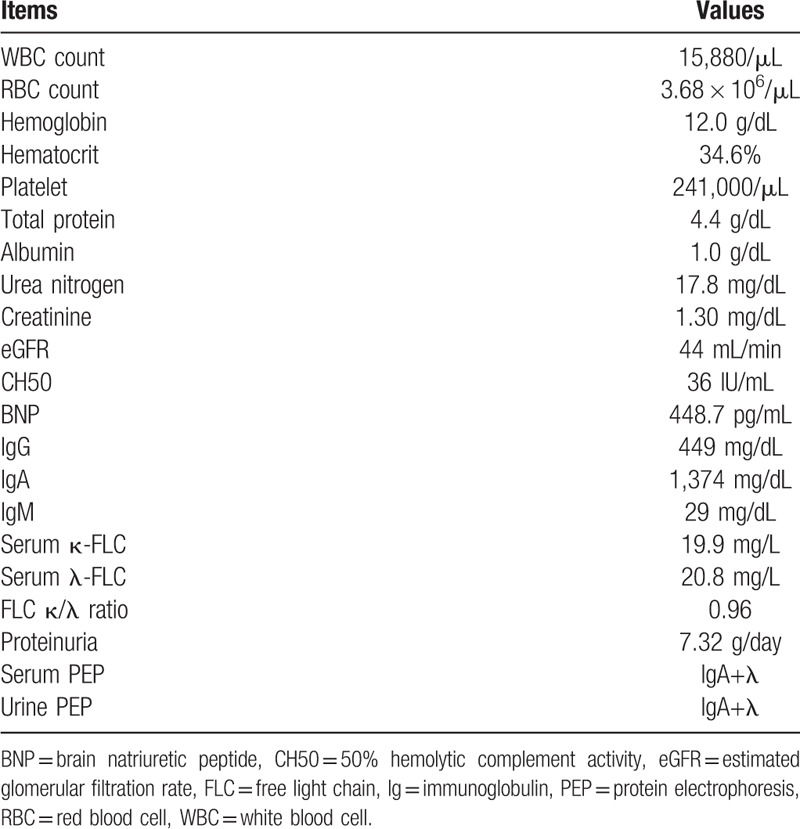
Initial laboratory findings on the day of admission.

**Figure 2:**
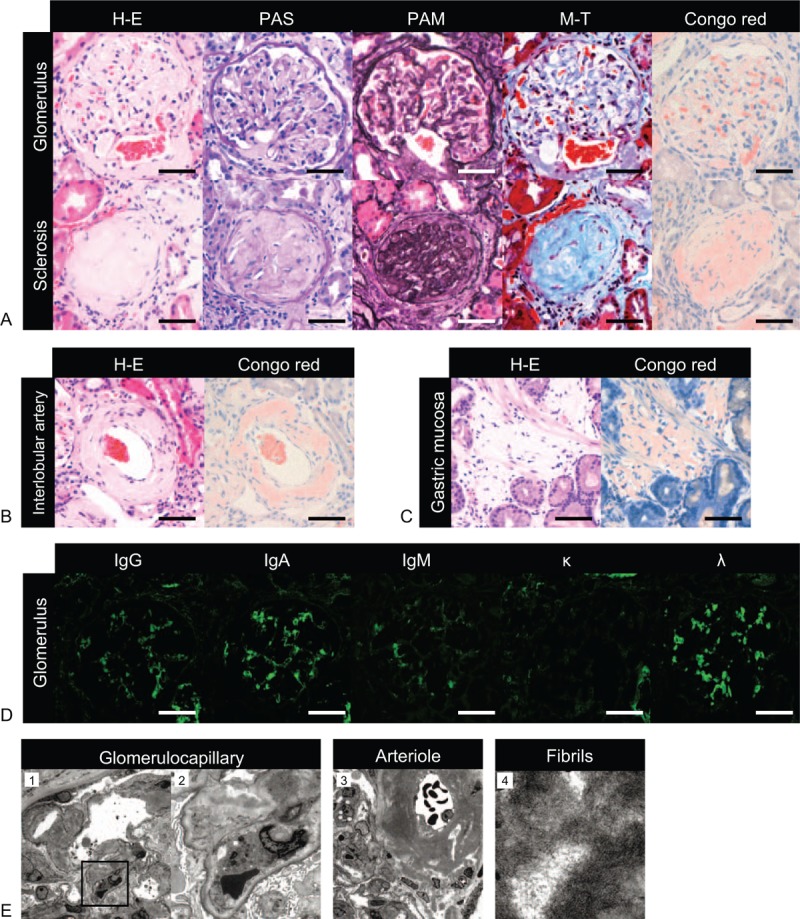
Percutaneous kidney biopsy demonstrates Periodic acid-Schiff (PAS)-positive hyalinization in most of the glomeruli (A) and surrounding vessels (B). Hematoxylin–eosin (H–E), periodic acid-methenamine-silver (PAM), Masson's trichrome (M-T) and Congo red stains are also shown. (C) Additionally, gastric mucosal biopsy by esophagogastroduodenoscopy demonstrates Congo red-positive amyloid deposition in the stromal regions. (D) Immunofluorescent staining reveals the deposition of immunoglobulin A and lambda in mesangial regions. Scale bars represent 50 μm. (E) Electron microscopy shows the deposition of randomly oriented amyloid fibrils. The magnifications are as follows: (1) ×1500; (2) ×5000; (3) ×1000; (4) ×30,000.

Percutaneous kidney and endoscopic gastric mucosal biopsy demonstrated Periodic acid-Schiff (PAS)-positive hyalinization in most of the glomeruli and surrounding vessel walls in the kidney (Fig. 2A and B) and submucosal stroma (Fig. 2C). These lesions were congophilic, which is considered a signature of systemic amyloidosis. Immunofluorescent (IF) staining revealed strong deposition of IgA and lambda (λ) chain in mesangial regions, but little IgG and IgM deposition, and no kappa (κ) light chain deposition (Fig. 2D). The amyloid deposits were composed of randomly oriented fibrils with a mean diameter of 10 nm on electron microscopy (Fig. 2E). Bone marrow aspiration biopsy showed little atypical plasma cell infiltration (<1%) and no evidence of myeloma.

The patient was referred to hematology for further treatment and moved to another hospital for the administration of chemotherapy using melphalan and dexamethasone. After 15-month follow-up from the initial diagnosis, the patient was still alive.

## 3 Literature analysis

### 3.1 Screening of the literature and the general characteristics of AHL amyloidosis

Clinical case reports and case series studies of AHL amyloidosis published between 2000 and 2017 were identified in PubMed (Medline database) using the search term, “heavy and light chain amyloidosis.” Identified publications were screened for inclusion using the following criteria: patient information, clinical history, laboratory data, and diagnostic methods were clearly described. The characteristics of AHL amyloidosis were assessed based on an analysis of the literature.

**Figure 3:**
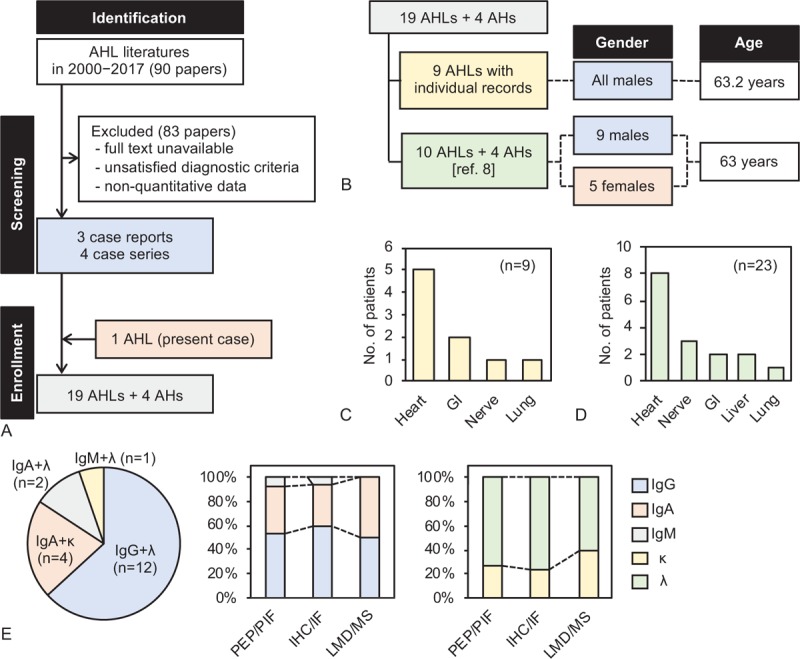
(A) The identification, screening, and study inclusion strategy is shown. (B) Nine cases of AHL amyloidosis with clear individual records and a group of 10 AHL and 4 AH amyloidosis cases from one series study^[8]^ were analyzed separately. The number of extra-renal complications is shown for 9 cases of AHL amyloidosis (C) and all 23 cases of AHL/AH amyloidosis (D). (E) Finally diagnosed amyloid proteins for all 19 cases of AHL amyloidosis are shown in the pie chart. The proportions of the type of amyloid protein detected by each procedure are shown in the bar graphs (*n* = 19).

A total of 7 publications (3 individual clinical case reports and 4 case series studies), with a total of 18 AHL amyloidosis cases, were selected for analysis (Fig. 3A).^[8–14]^ Among these, 15 cases were published from the United States, with most analyzed and reported by a single center, and one large series study comprised more than half of the cases.^[8]^ Nine of the cases including the present case had clear individual clinical data (Fig. 3B). All of these cases involved men and the mean age at diagnosis was 63.2 ± 4.98 years (mean ± standard deviation). The most common comorbidity was cardiovascular disease (*n* = 3, 33.3%) (e.g., hypertrophic obstructive cardiomyopathy and complete heart block,^[12]^ dilated cardiomyopathy,^[13]^ and congestive heart failure [present case]). Nephrotic syndrome (*n* = 2, 22.2%) and pneumonia (*n* = 2, 22.2%) were also observed. Among the extra-renal complications, cardiac involvement was the most common (*n* = 5, 55.6%), followed by gastrointestinal (GI) tract (*n* = 2, 22.2%), nerve (*n* = 1, 11.1%), and pulmonary involvement (*n* = 1, 11.1%) (Fig. 3C).

Additionally, among 16 AHL/AH amyloidosis cases reported by Nasr et al,^[8]^ an inseparable group of 10 AHL and 4 AH amyloidosis cases were selected for further analysis in the present study (Fig. 3B). There were 9 men (64.3%) and 5 women (35.7%), and the median age was 63 years (range, 50–77). These 14 cases were combined with the 9 cases of AHL amyloidosis described above and analyzed (*n* = 23). AHL/AH amyloidosis was 3.6-fold more common in men (*n* = 18, 78.3%) than in women (*n* = 5, 21.7%). Cardiac involvement remained the most frequent extra-renal complication (*n* = 8, 34.8%), followed by nerve (*n* = 3, 13.0%), GI tract (*n* = 2, 8.7%), liver (*n* = 2, 8.7%), and pulmonary involvement (*n* = 1, 4.3%) (Fig. 3D).

### 3.2 Applied diagnostic procedures for all 19 cases of AHL amyloidosis

Most of the AHL amyloidosis cases were diagnosed not only by conventional biochemical/pathological procedures such as serum and urine protein electrophoresis (PEP) or immunofixation (PIF), immunohistochemistry (IHC) and IF, but also by laser microdissection followed by mass spectrometry (LMD/MS). The major advantage of the proteomic approach with LMD/MS for amyloid typing is that LMD/MS is a single test that can be used for identifying various amyloid proteins simultaneously. In contrast, conventional methods involving the evaluation of tissue biopsies can be used to identify only individual target amyloid proteins of interest.^[15]^ Among the 19 cases, biochemical methods (PEP and/or PIF), pathological methods (IHC and/or IF), and LMD/MS were performed in 89.5% (*n* = 17), 94.7% (*n* = 18), and 57.9% (*n* = 11) of cases, respectively. Surprisingly, the combination of conventional methods was not inferior to LMD/MS alone for amyloid typing in the 11 cases diagnosed by both approaches (Table 2).

**Table 2:**
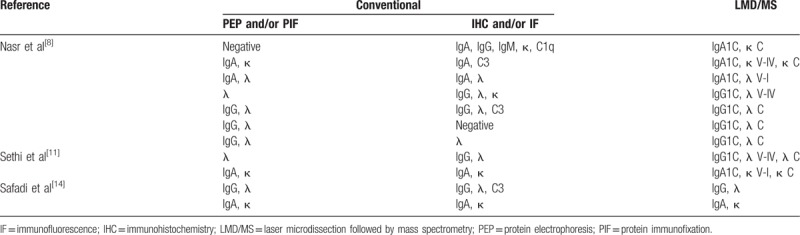
Diagnostic procedures used for amyloid typing.

The features of the detected amyloid fibrils in the 19 AHL amyloidosis cases are shown in Figure 3E. IgG+λ was the most frequent type (*n* = 12, 63.2%), followed by IgA+κ (*n* = 4, 21.1%), IgA+λ (*n* = 2, 10.5%), and IgM+λ (*n* = 1, 5.3%). There were no major differences between the detected types of amyloid proteins and diagnostic procedures. We were not able to assess the correlation between amyloid fibril type and patient survival due to a lack of complete clinical information.

## 4 Discussion

AHL and AL amyloidoses are always associated with an underlying plasma cell dyscrasia, ranging from low tumor burden conditions to those associated with multiple myeloma. While AL amyloidosis is the most common type of systemic amyloidosis, AHL amyloidosis is a rare condition accounting for only 5% of Ig-related amyloidosis.^[2,3]^ Our review of the current literature revealed that only 18 AHL amyloidosis cases have been reported in individual case reports and case series studies. Most of these cases (*n* = 15, 83.3%) were reported from the Mayo Clinic in the United States, and the remaining 3 cases were reported from Israel, India, and France.^[9,12,13]^ The present case is the first from Japan.

Among the 19 analyzed cases, we found that AHL/AH amyloidosis was 3.6-fold more common in men than in women. The age at diagnosis ranged from 50 to 77 years and mainly occurred in the early 60s. The global incidence of newly diagnosed multiple myeloma has been recently reported as 30,770 per year in total, with 16,400 male (53.3%) and 14,370 female cases (46.7%).^[16]^ Myeloma occurs slightly more frequently in men than in women, however, the difference is not very obvious. Moreover, sex differences in the clinical features of AHL amyloidosis remain unclear.

Amyloidosis often affects many organs and can present with a bewildering array of symptoms, depending on the organs involved. Although the initial symptoms are often nonspecific, the symptomatology reflects the impairment of the organs involved by the amyloidosis as the disease progresses. Certain clinical presentations require a diagnosis of amyloidosis to be considered: nephrotic range proteinuria, cardiac failure with left ventricular hypertrophy in the absence of hypertension or aortic valve disease, sensorimotor peripheral neuropathy without an obvious cause, hepatomegaly with a normal appearance on ultrasound or computed tomography imaging, and autonomic neuropathy.^[17]^

In this report, we described a rare case of systemic AHL amyloidosis with nephrotic syndrome and congestive heart failure. Renal involvement is one of the most common and critical complications in patients with Ig-related amyloidosis.^[4]^ Nephrotic syndrome, which is characterized by both heavy proteinuria and hypoalbuminemia, is the most common sign of primary amyloidosis of the kidneys. Since possible causes of nephrotic syndrome are very varied, renal biopsy is a key procedure to obtain the pathological diagnosis and to assess a primary cause. Cardiac amyloidosis is also a life-threatening complication of systemic amyloidosis. Electrocardiography and echocardiography are noninvasive and reproducible tools to investigate cardiac involvements at the initial screening of the disease. Low QRS voltage in the presence of the echocardiographic findings of increased myocardial wall thickness is highly suggestive of advanced amyloid heart disease.^[18]^ These critical findings were seen in the present case and brought a significant viewpoint of further prompt investigation and attentive follow-up of the patient.

Cardiac amyloidosis is a condition characterized by extracellular amyloid deposition that stiffens the heart without compensatory dilation, which results in increased wall stress and deteriorating cardiac function. Cardiac involvement is the second most frequent type of solid organ manifestation and is seen in up to 50% of patients with amyloidosis.^[19–21]^ In the present study, cardiac involvement was the most common extra-renal complication in AHL/AH amyloidosis (*n* = 8/23, 34.8%). In AL amyloidosis, survival depends mainly on the extent and severity of organ involvement, particularly the presence of cardiac involvement. Amyloid light chain cardiomyopathy is considered to be the most aggressive form of amyloid heart disease and is the principal reason for the poor prognosis. The natural history of AL amyloidosis is rapidly progressive, with a median overall survival of 18 months.^[19]^ Recent studies have shown that the prognosis of AL amyloidosis has improved with the development of new therapeutic strategies, such as stem cell and heart transplantation, increasing the median overall survival to 5 years or even more.^[4]^ Although AHL amyloidosis is considered to have less cardiac involvement than that in its much more frequent counterpart,^[8]^ the present study showed that cardiac involvement in AHL amyloidosis is greater than previously thought. Thus, the initial screening and follow-up of cardiac involvement are also important in AHL amyloidosis. This may improve the prognosis of patients through a reduction in cardiovascular events.

The LMD/MS-based proteomic approach is a novel diagnostic tool that enables the analysis of global protein expression patterns in the regions of interest and is used in the diagnosis and typing of amyloidosis, particularly when routine IHC and IF are equivocal.^[22,23]^ LMD/MS is considered to be much more precise than the conventional laboratory tests, however, it is limited in use and is not always accessible for every patient and clinician. Conventional biochemical and pathological approaches are often used to diagnose AHL amyloidosis in usual clinical settings. The combination approach with conventional methods is still promising in the diagnosis of AHL amyloidosis.

## 5 Conclusions

In summary, further investigation of the clinicopathological features of AHL amyloidosis is urgently needed. Especially, the prognosis of AHL amyloidosis must be elucidated to improve the strategies of diagnosis and treatment options for patients with this disease.

## Acknowledgments

We thank Drs Atsushi Nohara, Ayako Hoshi, Ayaka Tagahara-Yazaki, Satoru Sunaga, Haruno Nakajima, and Chie Tsunoda (Kidney Disease and Dialysis Center, Hidaka-kai Hidaka Hospital) for their aid in patient management; Drs Yuko Yamane, Yuka Yoshida, and Kinue Shimizu (Department of Pathology, Hidaka-kai Hidaka Hospital) for their aid in the pathological diagnosis. We also thank Taylor & Francis Editing Services (www.tandfeditingservices.com) for the English language review. The preliminary data of this study was presented at the 16th International Symposium on Amyloidosis (ISA 2018) in Kumamoto, Kumamoto, Japan.

## Author contributions

**Conceptualization:** Yukihiro Otaka, Yoichi Nakazato, Takaaki Tsutsui, Jun’ichi Tamura.

**Formal analysis:** Yukihiro Otaka.

**Investigation:** Yukihiro Otaka, Yoichi Nakazato, Takaaki Tsutsui.

**Supervision:** Yoichi Nakazato, Takaaki Tsutsui, Jun’ichi Tamura.

**Writing – original draft:** Yukihiro Otaka.

**Writing – review & editing:** Yukihiro Otaka, Yoichi Nakazato, Takaaki Tsutsui, Jun’ichi Tamura.

Yukihiro Otaka orcid: 0000-0002-9162-7188.
